# Supplementary material for: Effects of High-Linear-Energy-Transfer Heavy Ion Radiation on Intestinal Stem Cells: Implications for Gut Health and Tumorigenesis
Source: Cancers (Basel). 2024 Oct 4;16(19):3392. doi: 10.3390/cancers16193392 (PMC11475725; doi:10.3390/cancers16193392)
Supplement: Supplementary file 1 [file cancers-16-03392-s001.zip › cancers-3220522-supplementary.pdf]

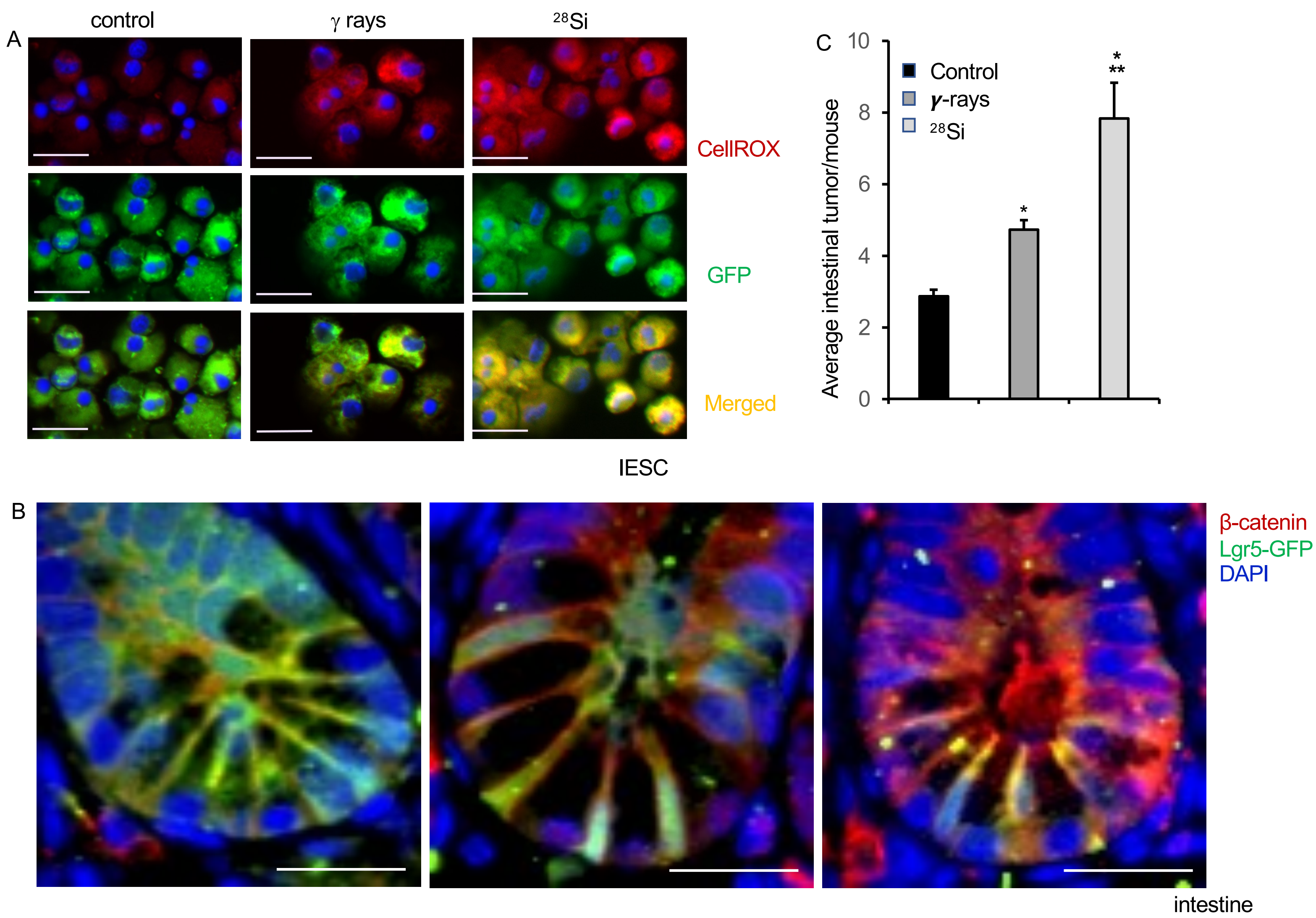

Figure S1: HZE induces persistent oxidative stress in Lgr5-GFP positive intestinal stem cells. (A) Sorted Lgr5-GFP ISC were stained for RellROX deep red to assess the oxidative stress. Stained cells were fixed, cytospin onto a glass slide, and visualized under a microscope. Nuclei were co-stained with DAPI. Representative images show increased CellROX stain in irradiated samples at a 2-month time. (B) Immunofluorescent co-staining of β-catenin and Lgr5 -GFP in intestine 2 months post irradiation. (C) Average tumor count per mouse in *Lgr5^{+}Apc$^{1638N/+}$* mice mice. Scale bar, 20 μm. *, significant relative to control; **, significant relative to γ-rays. Statistical significance is set at $P < 0.05$, and error bars represent mean ± SEM.

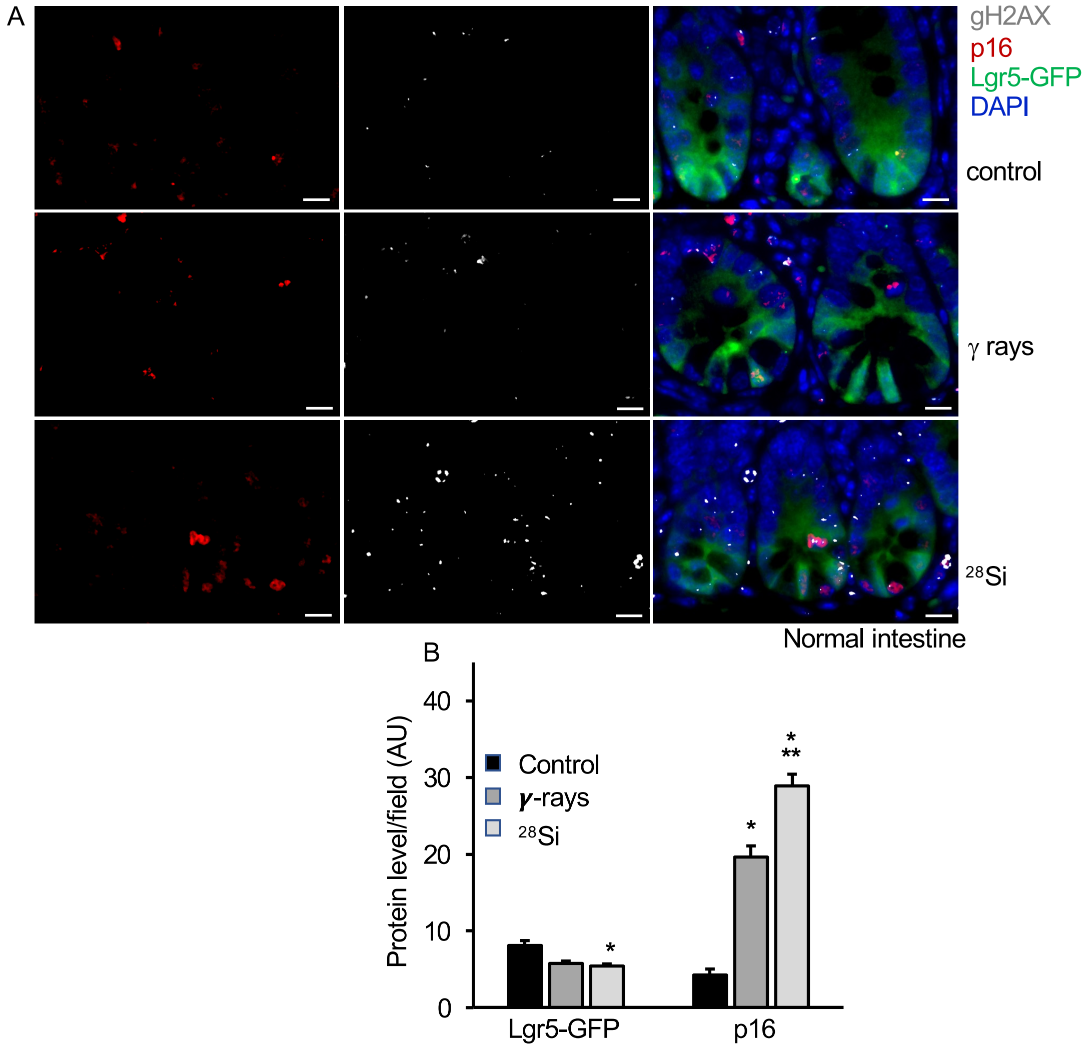

Figure S2: ^{28}Si exposure leads to persistent increased DNA damage and senescence after 5 months of radiation in non-tumor normal intestine from *Lgr5^{+}Apc$^{1638N/+}$* mice. Adjacent normal intestinal sections were co-stained with p16, γH2AX, and Lgr5-GFP to detect DNA damage response and senescence after 5 months of radiation exposure. (A) After radiation exposure, representative fluorescent images show increased p16 and γH2AX expression in non-tumor tissue sections. (B) γH2AX foci were counted and represented graphically, showing higher DNA damage response in irradiated samples relative to control. Graphical representation of Lgr5-GFP or p16 fluorescent intensity in the non-tumor intestine with or without radiation. Nuclei were visualized using DAPI. Scale bar, 10 µm. *, significant relative to control; **, significant relative to γ-rays. Statistical significance is set at $P < 0.05$, and error bars represent mean ± SEM.

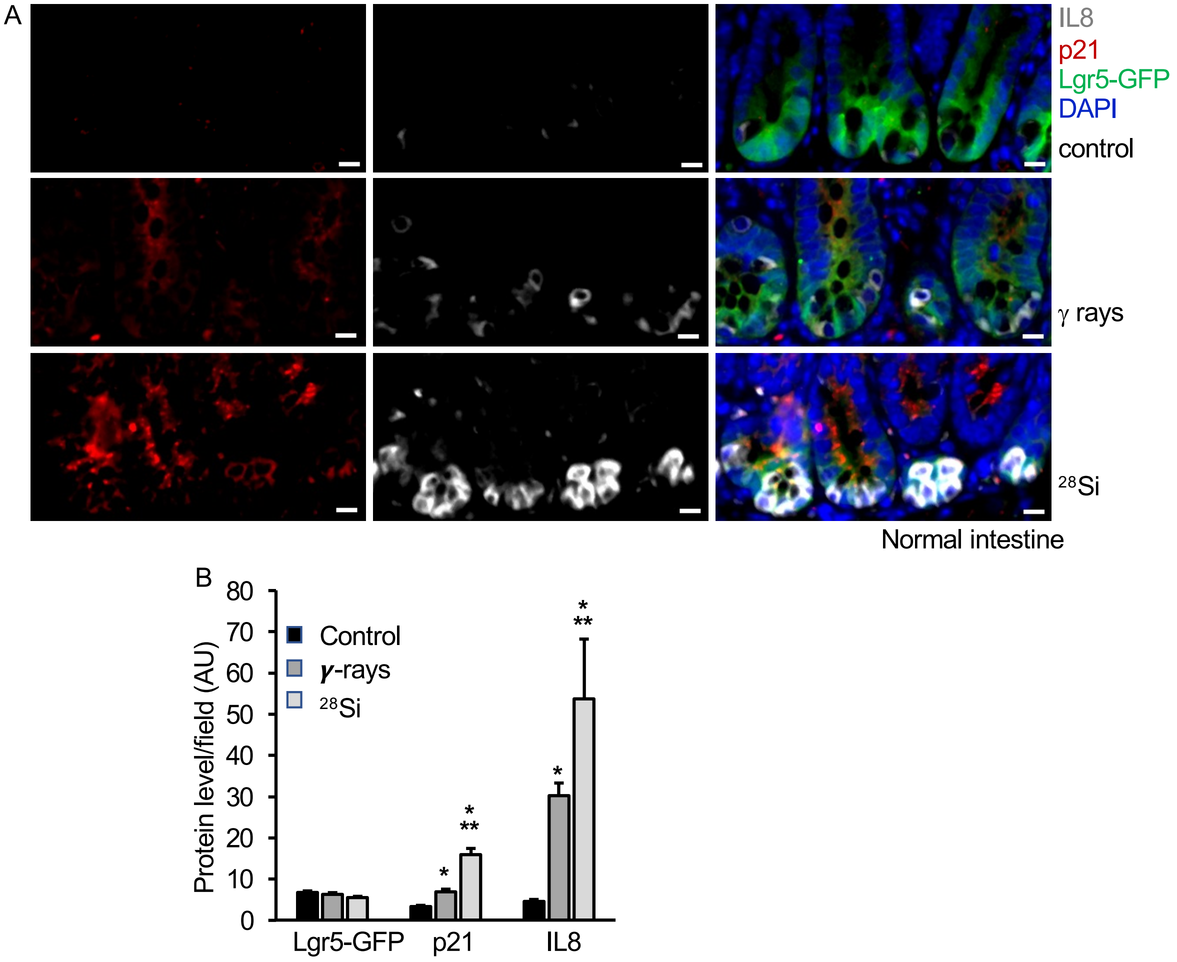

Figure S3: ^{28}Si exposure leads to increased senescence and SASP acquisitions after 5 months of radiation in non-tumor normal intestine derived from *Lgr5^{+}Apc$^{1638N/+}$* mice. To detect senescence and secretory phenotype, in non-tumor normal intestinal sections were co-stained with p21, IL8, and Lgr5-GFP. Signals were detected, and images were captured under fluorescent microscopy. (A) Representative fluorescent images show enhanced expression of IL8 and p21 expression in intestinal non-tumor normal tissue. (B) The fluorescent intensity of IL8, p21, and Lgr5-GFP was quantified and represented as a bar graph. Nuclei were visualized using DAPI. Scale bar, 10 µm. *, significant relative to control; **, significant relative to γ-rays. Statistical significance is set at $P < 0.05$ and error bars represent mean ± SEM.

Table S1: List of SASP and GI function primers used for qRT-PCR

S.No.	Gene	Primers
1	ATF5	Forward 5’-gggtcattttagctctgtgagagaa; Reverse 5’-atttgtgcccataaccccctaga
2	Bax	Forward 5’-atgcgtccaccaagaagctga; Reverse 5’-agcaatcatcctctgcagctcc
3	CD40	Forward 5’-gcagtgtgttacgtgcagtg; Reverse 5’-ctgtgcagtggcttgtcagt
4	Cyclind1	Forward 5’-ttgactgccgagaagttgtg; Reverse 5’-ccacttgagcttgttcacca
5	CyclinD2	Forward 5’- caccgacaactctgtgaagc; Reverse 5’- tgctcaatgaagtcgtgagg
6	Cxcl1	Forward 5’-ctgggattcacctcaagaacatc; Reverse 5’-cagggtcaaggcaagcctc
7	Cxcl2	Forward 5’-ccaaccaccaggctacagg; Reverse 5’-gcgtcacactcaagctctg
8	Cxcl5	Forward 5’-tgcgttgtgtttgcttaaccg; Reverse 5’-agctatgacttccaccgtagg
9	Faim2	Forward 5’-ctcgagagaagacatcatgacc; Reverse 5’-ttctctccatttgcctggtg
10	IL1β	Forward 5’-gcactacaggctccgagatgaac; Reverse 5’-ttgtcgttgcttggttctccttgt
11	IL6	Forward 5’-caagaaagacaaagccagagtc; Reverse 5’-gaaattggggtaggaaggac
12	Lpo	Forward 5’-tgaccttgctccagactgc; Reverse 5’-ttgacccagaccttgacctc
13	Opg	Forward 5’-atgaacaagtggctgtgctg; Reverse 5’-tcacacaggagctgatgacc
14	p16	Forward 5’-gggtttcgcccaacgccccga; Reverse 5’-tgcagcaccaccagcgtgtcc
15	P19arf	Forward 5’-gtcacacgactgggcgatt; Reverse 5’-gactccatgctgctccagat
16	p21	Forward 5’-tccacagcgatatccagaca; Reverse 5’-agacaacggcacactttgct
17	PLAT	Forward 5’-agttcctgctgggtgctgtc; Reverse 5’-cggggaccaccctgtatgtt
18	PLA2G2a	Forward 5’-taagacaggaaagagagctgagc; Reverse 5’-gtaccacatccacttttctccag
19	PTGES	Forward 5’-agcacactgctggtcatcaa; Reverse 5’-tccacatctgggtcactcct
20	Sox17	Forward 5’-tgaaatatggcccactcaca; Reverse 5’-ctgtcttccctgtcttggttg
21	Tlr1	Forward 5’-ggacctacccttgcaaacaa; Reverse 5’-tatcaggaccctcagcttgg
22	Troy	Forward 5’-cgctgccattctcttcctac; Reverse 5’-tcgatccttgaattcctgct
23	Gapdh	Forward 5’-gtggagtcatactggaacatgtag; Reverse 5’-aatggtgaaggtcggtgtg
24	Scl2a2	Forward 5’-cgaatttatccagcagcacaag; Reverse 5’-ggccatcaacatgatcttcac
25	Scla2a5	Forward 5’-tgatatagggcatccaggagat; Reverse 5’-tgaccatcctcacgatctttg
26	Scla5a1	Forward 5’-gataaaagaccccaccagcat; Reverse 5’-cttgggcctggatatatacttgg
27	Cck	Forward 5’-ccggtcacttattctatggct; Reverse 5’-cactgctagcgcgatacatc
28	Gip	Forward 5’-gcaagctaaggtcagagtcc; Reverse 5’-gatgatgtgctgagagacctt
29	Scl9a3	Forward 5’-cctcatgctctgaagttcca; Reverse 5’-cggccaagctgtacaagag
30	Scl27a4	Forward 5’-gatgatgcgacccacca; Reverse 5’-catcctgtcctttgtgtaccc
31	Npc1	Forward 5’-ccctccaaatttcgtaagtgtg; Reverse 5’-gcagccatataacgagagcat
32	Npc1l1	Forward 5’-gctagtttctcctccagtacca; Reverse 5’-ctcaacctcctgatcaccttg

Table S2: Oxidative stress PCR array in Lgr5-GFP positive cells 60-day post radiation exposure

Gene Symbol	Gene Name	γ rays Fold change ± SE	^{28}Si Fold change ± SE
Sod1	Superoxide dismutase 1, soluble	4.99±0.44	5.62±0.23
Prdx6	Peroxiredoxin 6	4.91±0.31	6.81±0.31
Noxo1	NADPH oxidase organizer 1	4.86±0.18	4.06±0.23
Gpx2	Glutathione peroxidase 2	4.78±0.23	3.32±0.28
Epx	Eosinophil peroxidase	2.94±0.39	3.13±0.32
Ercc6	Excision repair cross-complementing rodent repair deficiency, complementation group 6	3.78±0.22	4.07±0.25
Gpx5	Glutathione peroxidase 5	2.92±0.39	4.14±0.34
Park7	Parkinson disease (autosomal recessive, early onset) 7	2.86±0.25	2.53±0.23
Ercc2	Excision repair cross-complementing rodent repair deficiency, complementation group 2	2.69±0.23	2.83±0.23
Nqo1	NAD(P)H dehydrogenase, quinone 1	2.62±0.23	2.87±0.32
Sod2	Superoxide dismutase 2, mitochondrial	2.6±0.26	2.68±0.19
Ccs	Copper chaperone for superoxide dismutase	2.6±0.23	2.81±0.25
Il19	Interleukin 19	2.51±0.41	2.44±0.04
Xpa	Xeroderma pigmentosum, complementation group A	2.46±0.26	2.49±0.22
Gss	Glutathione synthetase	2.35±0.19	2.60±0.19
Als2	Amyotrophic lateral sclerosis 2 (juvenile) homolog (human)	2.20±0.19	2.55±0.23
Hmox1	Heme oxygenase (decycling) 1	2.01±0.42	3.00±0.28
Cygb	Cytoglobin	-2.74±0.31	2.44±0.22
Fancc	Fanconi anemia, complementation group C	7.61±0.23	8.29±0.24
Atr	Ataxia telangiectasia and rad3 related	7.25±0.33	3.05±0.26
Slc38a1	Solute carrier family 38, member 1	2.7±0.24	2.36±0.30
Ift172	Intra-flagellar transport 172 homolog (Chlamydomonas)	2.29±0.22	2.67±0.22
Duox1	Dual oxidase 1	2.3±0.20	2.29±0.22
Txrnd3	Thioredoxin reductase 3	4.57±0.29	4.48±0.24
Prdx4	Peroxiredoxin 4	2.19±0.23	2.49±0.21
Apc	Adenomatosis polyposis coli	2.08±0.22	2.25±0.22
Ehd2	EH-domain containing 2	-2.07±0.39	-2.53±0.12
Mpo	Myoglobin	-14.86±0.31	-2.47±0.31
Fmo2	Flavin containing monooxygenase 2	-2.29±0.28	-2.42±0.24
Aox1	Aldehyde oxidase 1	-5.87±0.14	-2.67±0.31

Table S3: Expression of SASP and SIR genes in Lgr5-GFP cells 60-day post radiation expression

Gene	Functions	γ rays Fold change ± SE	^{28}Si Fold change ± SE
ATF5	Tumor suppressor, enhances radio resistance	1.09±0.11	1.44±0.31
Bax	Proapoptotic signaling	-1.42±0.03	-1.61±0.06
CD40	Induce ROS and NO level in cell with activated CD40	-1.42±0.9	1.08±0.18
Cyclind1	G1/S phase transition and entry into the S-phase	1.47±0.25	2.23±0.25
CyclinD2	Regulates cell-cycle during G (1)/S transition.	1.22±0.27	1.74±0.24
Cxcl1	Mitogenic, chemo-attractant for neutrophil	1.22±0.06	1.08±0.18
Cxcl2	Immunoregulatory and inflammatory processes	1.95±0.17	1.49±0.09
Cxcl5	Stimulates chemotaxis of neutrophil	1.09±0.24	1.74±0.24
Faim2	Fas apoptotic inbibitory molecule 2	1.05±0.26	2.07±0.39
IL1β	Mediator of inflammatory response, involved in a cell proliferation, differentiation, and apoptosis	2.94±0.5	3.3±0.2
IL6	Inflammatory response cytokine	2.73±0.2	3.4±0.2
Lpo	A oxidoreductase, acts as a natural antibacterial agent	-1.26±0.19	1.38±0.37
Opg	TNF signaling	-1.09±0.09	1.21±0.12
p16	Cyclin dependent kinase inhibitor	1.90±0.2	2.6±0.3
P19arf	Induces cell cycle arrest in G1 and G2 phases	1.87±0.3	3.5±0.1
p21	Cyclin dependent kinase inhibitor	1.46±0.2	2.0±0.2
PLAT	Secreted serine protease that converts proenzyme plasminogen to plasmin,	-1.01±0.32	1.88±0.54
PLA2G2a	Inflammatory response and tissue regeneration, regulate phospholipid metabolism in bio membrane	-1.26±0.09	2.15±0.27
PTGES	Prostaglandin E synthase, induced by IL1beta, TP53. Medi pain during acute inflammatory response	1.08±0.26	2.55±0.19
Sox17	Transcriptional regulator of protein involved in development and in the determination of cell fate	1.10±0.25	1.91±0.14
Tlr1	pathogen recognition and activation of innate immunity.	-1.13±0.43	1.57±0.13
Troy	Activates JNK signaling	1.22±0.35	1.37±0.18
